# Repertory of Characteristics

**Published:** 1885-12

**Authors:** 


### REPERTORY OF CHARACTERISTICS.

We expect to commence printing this repertory about January 1st, and hope to have it completed early in June. We feel much gratified at the interest shown in this work, and hope to make a useful and reliable book—such an one as will be a valuable assistant to us all in our practice.

